# Small extracellular vesicles: messengers at the service of breast cancer agenda in the primary and distant microenvironments

**DOI:** 10.1186/s13046-025-03471-y

**Published:** 2025-07-21

**Authors:** Angela Galardi, Valentina Fogazzi, Claudia Tottone, Marta Giussani, Serenella M. Pupa, Giulia Cosentino, Marilena V. Iorio

**Affiliations:** 1 Microenvironment and Biomarkers in Solid Tumors Unit, Department of Experimental Oncology, Fondazione IRCCS Istituto Nazionale dei Tumori, Milan, Italy; 2 SC Laboratory Medicine, Department of Advanced Diagnostic and Services, Fondazione IRCCS Istituto Nazionale dei Tumori, Milan, Italy

**Keywords:** Small extracellular vesicles, Breast cancer, Microenvironment, Immune cells, Stroma cells, MicroRNAs

## Abstract

Breast cancer (BC) remains a leading cause of cancer-related mortality in women, with complex mechanisms driving its initiation, progression, and resistance to therapy. In recent years, the tumor microenvironment (TME) has gained attention for its critical role in shaping tumor behavior, where small extracellular vesicles (small EVs) have emerged as key mediators of intercellular communication. These vesicles carry a diverse cargo of proteins, lipids, DNA, and various non-coding RNAs—such as miR-21, miR-155, and miR-1246—mirroring the molecular status of their originating cells. This review highlights the roles of small EVs in immune modulation, stromal remodelling, and metastatic niche formation, emphasizing their contribution to therapy resistance and immune evasion. We discuss recent updates on EV biogenesis, characterisation and isolation techniques, such as ultracentrifugation, immunoaffinity and microfluidic systems. We also critically evaluate their potential for clinical application and how well they conform to the MISEV2023 guidelines. Furthermore, we examine small EVs as diagnostic tools in liquid biopsies and compare them with conventional methods such as mammography and tissue biopsies. We also discuss organotropism mediated by small EV cargo (e.g., integrins *α6β4*,* αvβ5*) and the diagnostic potential of protein and lipid signatures (e.g., PD-L1, CD63, and exosomal lipidomics). Therapeutically, we explore engineered small EVs for drug delivery, gene modulation, and immune activation, addressing challenges of targeting efficiency, in vivo stability, immunogenicity, and clinical scalability. The review discusses ongoing clinical trials involving small EVs in BC and highlights key translational gaps between preclinical advances and clinical implementation. Finally, we explores how integrating artificial intelligence, single-cell transcriptomics, and multi-omics approaches can help overcome major challenges such as small EV heterogeneity and tracking limitations. Crucially, this integration enables a more tailored understanding of each patient’s tumor biology, reducing therapeutic failures by guiding more personalized and effective treatment strategies. Overall, small EVs represent a transformative tool in precision oncology, contingent on resolving key challenges in their clinical translation.

## Background

**Fig. 1:**
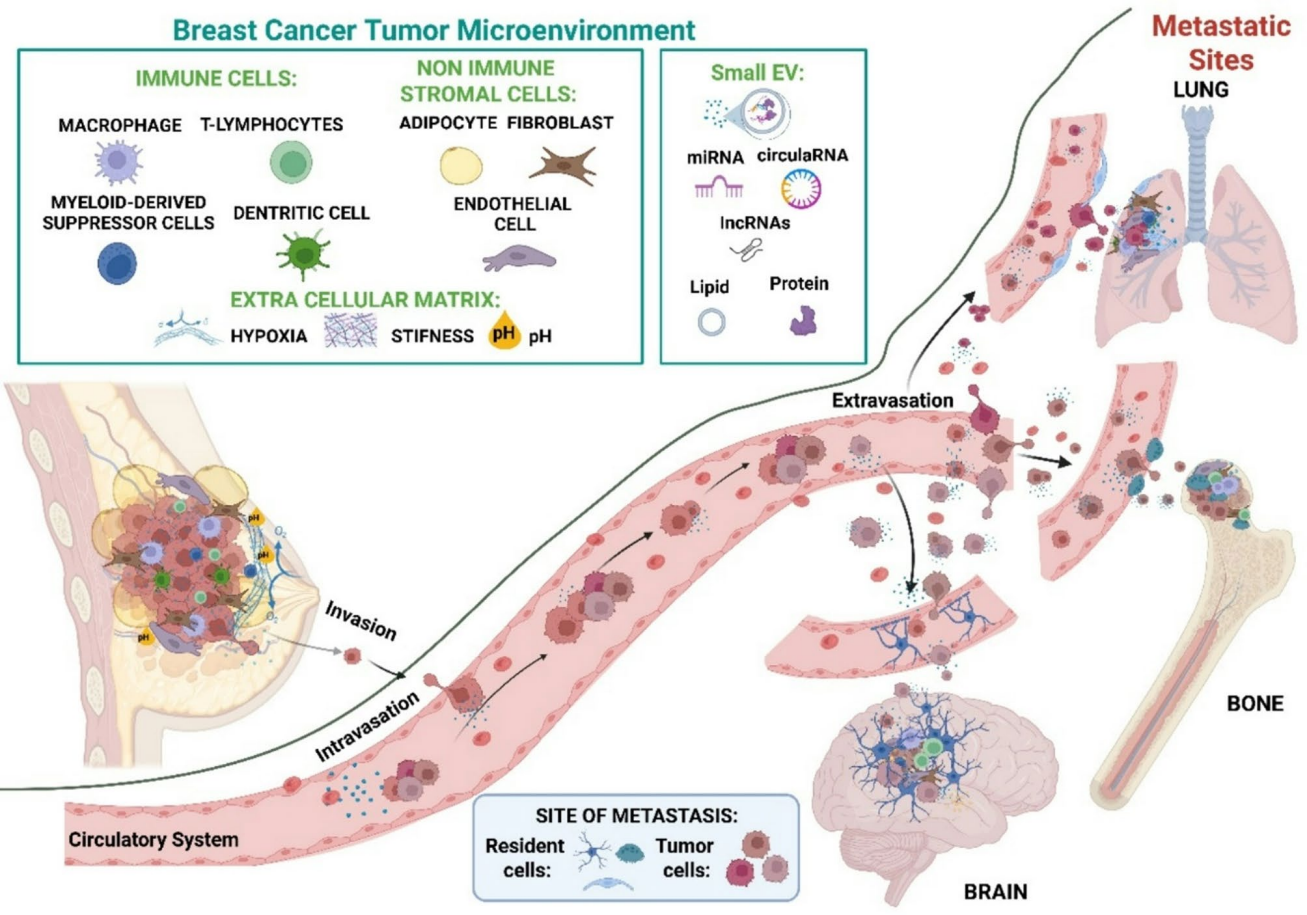
Role of *small EVs* in breast cancer (BC) progression and metastasis. The involvement of small EVs in the metastatic cascade of BC. Within the primary tumor microenvironment, tumor-derived small EVs mediate crosstalk with both immune (macrophages, dendritic cells, T lymphocytes, myeloid-derived suppressor cells) and non-immune stromal cells (fibroblasts, endothelial cells, adipocytes), as well as the extracellular matrix. Small EVs facilitate tumor progression by promoting invasion and intravasation of cancer cells into the circulatory system. These vesicles also contribute to the formation of pre-metastatic niches at distant sites (lung, bone, brain) by transferring oncogenic cargo—miRNAs, proteins, circular RNAs, lncRNAs, and lipids—to resident cells, thus supporting extravasation and colonization. This highlights the central role of small EVs in preparing metastatic sites and modulating the tumor microenvironment at both primary and distant locations (Created with BioRender.com)

Breast cancer (BC) is the most common malignancy in women and is still a major cause of death, and its incidence and mortality rates are on the rise [1]. BC is a complex and multifactorial disease, classified based on the expression patterns of three key receptors: estrogen receptor (ER), progesterone receptor (PR), and human epidermal growth factor receptor 2 (HER2). These expression profiles and the proliferation rate define four major BC subtypes: luminal A, luminal B, HER2-positive, and triple-negative breast cancer (TNBC) [2]. Despite advances in early detection methods and therapeutic interventions, there remains a critical need for new biomarkers to better predict disease progression, response to therapy and overall survival [3]. This complexity is not only due to cancer cells themselves. It is also profoundly influenced by the tumor surrounding microenvironment (TME), which plays a central role in cancer initiation, progression and response to treatment [4]. The TME is the ecosystem that surrounds and feeds a tumor mass inside the body [5]. It includes normal, neoplastic and immune cells, the extracellular matrix, blood vessels, other stromal cells, signaling messengers and various biochemical and mechanical factors. It is a complex and continuously evolving entity and plays a critical role in promoting tumor growth and progression [6]. The composition of the TME varies between tumor types and changes during tumor progression [7]. In this complex scenario, understanding how cells communicate with each other and the messages they exchange is crucial. Recent studies have highlighted the multifaceted roles of small EVs (30–200 nm in size), and their diverse and heterogeneous cargo, including microRNAs (miRNAs), long non-coding RNAs (lncRNAs), lipids, proteins and DNA. Small EVs and their content from TME-resident cells facilitate interactions between the tumor and its microenvironment, for example immune cells, regulating different tumor biological processes [8]. Indeed, the ability of tumors to hijack or alter the immune response by establishing an immunosuppressive niche represents a major barrier to effective anti-cancer treatments. Additionally, the cell interactions occurring within the TME could potentially define specific tropism for metastasis to particular sites. Notably, both tumor cells and their EVs may reflect the underlying molecular heterogeneity of BC, which adds further complexity to these interactions. Therefore, a deeper understanding of these multifaceted interactions may lead to designing novel therapeutic strategies aimed at reprogramming the altered resident stromal cells while also acting at the metastatic sites (Fig. 1).

## The tumor microenvironment: composition and physicochemical properties

### Cellular components of the TME

The TME is a highly dynamic and complex system that can either promote or inhibit anti-tumor activities [9]. It comprises various cellular and non-cellular components, including immune cells, stromal cells, and the extracellular matrix (ECM), which provides both structural support and signaling cues [10]. The immune system, the body’s natural defense mechanism, protects against pathogens, infections, and diseases. It is a dynamic network of cells, tissues, and organs that operate through two main defense lines: innate immunity and adaptive immunity [11]. Both play critical roles in the development and progression of BC [12]. Innate immune cells in the TME include macrophages, dendritic cells (DCs), myeloid-derived suppressor cells (MDSCs), natural killer (NK) cells, mast cells, and granulocytes such as neutrophils, basophils, and eosinophils. These cells contribute to the early response to tumor development but can also be reprogrammed by cancer cells to support tumor progression [13]. Adaptive immune cells, such as T and B cells, also infiltrate the TME. T cells include subsets such as T helper cells (Th), cytotoxic T lymphocytes (CTLs), and regulatory T cells (Tregs), which play crucial roles in modulating immune responses. Natural killer T (NKT) cells bridge innate and adaptive immunity and are involved in tumor surveillance [14]. Non-immune stromal cells regulate multiple processes in the TME, including tumor metabolism, proliferation, invasion, immune evasion, stemness, and treatment resistance [15]. These include vascular cells (endothelial cells and pericytes), fibroblasts, adipocytes, and mesenchymal stem cells [16]. The main stromal component in BC is represented by cancer-associated fibroblasts (CAF) [17]. CAFs are a heterogeneous group of tumor stromal cells. They are morphologically fibroblast-like [18]. They are often activated and can secrete extracellular matrix components, growth factors, and cytokines that promote tumor cell proliferation, invasion, and angiogenesis. CAFs can also help in the remodeling of the ECM, creating a more favorable environment for tumor spread. Another key stromal component in BC is the adipocyte. In hormone-responsive tumors in particular, surrounding adipocytes can release various adipokines and cytokines that support cancer cell growth and migration by meeting their metabolic demands. Importantly, the cellular composition of the TME is itself highly heterogeneous, varying not only between tumor types but also within individual tumors over time. This heterogeneity contributes to diverse patterns of tumor behavior and therapy response and represents a challenge for both diagnosis and therapeutic targeting.

### Physicochemical properties of the TME

In this “dense” and busy environment, we cannot consider the chemical and physical properties of the TME apart from the cellular component. Matrix composition and rigidity, extracellular pH, and other physicochemical assets have also been identified as modulators of tumor cell properties [19]. Hypoxia, defined as low oxygen levels [20], is a critical factor in several solid tumors, and in particular in BC, it has a major impact on the TME and promotes tumor growth, metastasis and resistance to therapy [21]. Under hypoxic conditions, TME in BC shows immunosuppression [22] and metabolic reprogramming [23]. Changes in the mechanical properties, including stiffness, of the ECM and surrounding tissue influences tumor behavior [24]. TME rigidity is a critical physical and biochemical property that plays a significant role in BC progression [25]. In BC, matrix stiffness can affect the proliferation, epithelial-to-mesenchymal transition (EMT), metastasis, invasion, immune evasion, stemness, and drug resistance, thus further promoting fibrosis and tumor progression [26]. Chemically, the TME is enriched with growth factors, cytokines, and proteolytic enzymes secreted by tumor and stromal cells, creating a pro-tumorigenic and immunosuppressive environment. Acidification, a hallmark of the TME, results from an enhanced glycolysis rate in cancer cells (the Warburg effect), which produces high lactate levels and lowers the extracellular pH [27] by globally rewiring cancer cell metabolism [28].

## Small evs: biogenesis and cargo

Small EVs are membrane-bound nanostructures, typically less than 200 nm in diameter, secreted by a wide range of cell types under both physiological and pathological conditions. They play a vital role in intercellular communication by transporting molecular cargo—including proteins, lipids, RNAs, and DNA—that modulates the behavior of recipient cells [29]. Small EVs are crucial modulators of the TME, influencing processes such as immune evasion, angiogenesis, and metastasis. In BC, they are actively secreted by both tumor cells and TME-associated cells, significantly contributing to these same pathological mechanisms [30, 31]. These vesicles not only are present in greater abundance compared to those from healthy cells but also carry a distinct cargo profile that reflects the molecular identity and status of their cell of origin [32]. Small EVs are generated through different biogenetic pathways, including the endosomal route and direct budding from the plasma membrane—mechanisms that contribute to their structural and functional heterogeneity. According to the MISEV2023 guidelines, the term “exosome” should be reserved for vesicles of endosomal origin, formed via inward budding of multivesicular bodies (MVBs) and released upon fusion with the plasma membrane. In all other cases, including when the origin is uncertain, the broader term “small EVs” is preferred to avoid misclassification [33]. *Exosome* formation through the endosomal pathway involves the Endosomal Sorting Complex Required for Transport (ESCRT). This process begins with ESCRT-0 binding to ubiquitinated receptors on early endosomes, initiating membrane invagination. ESCRT-I and ESCRT-II then assist in vesicle budding, while ESCRT-III mediates final vesicle scission. The resulting MVBs may either fuse with lysosomes for degradation or with the plasma membrane to release *exosomes* into the extracellular space [34, 35]. Among the most studied components of small EVs there are microRNAs (miRNAs), small non-coding RNAs (~ 22 nucleotides) that regulate gene expression at the post-transcriptional level. Their production begins in the nucleus, where primary transcripts (pri-miRNAs) are processed by the Drosha enzyme into precursor miRNAs (pre-miRNAs). These pre-miRNAs are exported to the cytoplasm by Exportin-5, where they are further processed by Dicer into mature miRNA duplexes. One strand of the duplex is incorporated into the RNA-induced silencing complex (RISC) to guide gene silencing, while the other strand is typically degraded. The incorporation of miRNAs into small EVs is not random but highly selective, involving multiple molecular mechanisms. For example, the enzyme nSMase2 facilitates miRNA packaging through ceramide-mediated vesicle formation. RNA-binding proteins such as hnRNPs recognize specific sequence motifs—like GGAG or GUGCU at the 3′ end of the miRNA—that promote sorting into vesicles. Additionally, 3′-uridylation and association with Argonaute 2 (Ago2) have been shown to influence miRNA loading, highlighting the importance of both sequence and structural features in the selection process. However, the complete sorting process remains unclear [36, 37]. This concept of selective sorting was further clarified by García-Martin et al., who showed that miRNA profiles in *exosomes* are not random but cell type–specific, driven by dedicated sequence motifs. Using five metabolically important cell types and miRNA profiling, *Garcia-Martin et al.;* showed that the population of miRNAs released in *exosomes* is peculiar for each tissue type and miRNA sequence analysis shows that this strongly correlates with the presence of *exosomes* export sequences (EXOmotifs) versus cell retention sequences (CELLmotifs). Indeed, some EXOmotifs are enriched up to 80-fold in *exosomal*-miRNAs. The introduction of miRNA sorting motifs can drastically improve the transfer or cellular retention of *exosomal-*miRNAs, leading to increased transfer to target cells and reduced expression of target genes. They also discovered that Alyref and Fus are two proteins involved in EXOmotif sorting with the highest exosomes enrichment [CGGAG] [38].

The molecular cargo of small EVs is definitely not restricted to miRNAs alone; they can also contain a wide range of other potential components, including proteins, lipids, enzymes, and signaling molecules, all of which can function remotely from the cells that produce small EVs [39]. It is clear that the horizontal or paracrine transfer of these chemicals is associated with the growth, invasion, and metastatic spread of cancer cells when they reach the targeted recipient cells in the TME or other distant metastatic niches [40]. Small EVs are enriched in a conserved set of proteins that play essential structural and functional roles. Adhesion-related tetraspanin proteins such as CD63, CD9, and CD81 are commonly used as EV markers. Components of the endosomal sorting complex involved in EV biogenesis pathways, such as TSG101 and Alix, are also frequently present. Other EV proteins are specifically selected and enriched, suggesting an active sorting mechanism by tumor cells. These proteins are divided into different categories, according to their function. Some proteins are involved in the immune response, creating a favorable environment for tumor growth, such as PD-L1 [41]. On small EVs, we can find integrins, transmembrane proteins playing a crucial role in determining the preferential site of metastasis. This means that small EVs released from the primary tumor, depending on the integrins they expose on their surface, preferentially target specific organs, where they modify the microenvironment to prepare it for the arrival of tumor cells [42]. Lipid components in small EVs as cholesterol, ceramide, phosphatidylserine and sphingomyelin influence vesicle stability and fusion with recipient cells, playing a role in intracellular signaling [43]. Long non-coding RNAs (lncRNAs) have also emerged as important EV cargo in BC. These molecules contribute to tumor progression, immune modulation, and therapy resistance. LncRNAs, which are known to regulate cytoskeletal dynamics and ECM remodeling, are also selectively packaged into BC–derived small EVs. Once transferred to recipient stromal or endothelial cells, they are implicated in EMT and enhance tumor invasiveness by modulating actin filaments and matrix metalloproteinase activity. This interplay between lncRNAs and cytoskeleton/ECM crosstalk via small EVs is increasingly recognized as a driver of metastatic behavior in BC [44]. Moreover, small EV biogenesis and cargo composition are not only governed by intrinsic cellular mechanisms, but they are also influenced by various microenvironmental factors. In particular, conditions such as hypoxia, increased stiffness, and acidification have been shown to actively contribute to these processes, further shaping the small EVs profile and function [45]. These factors not only regulate tumor growth, but also influence the uptake and release of small EVs, shaping intercellular communications within the TME and distant metastatic niches. Given their significant role in tumor progression, small EVs present a promising target for therapeutic interventions aimed at reprogramming the TME and altering the course of cancer development. Furthermore, the crosstalk between tumor cells and the TME not only modulates local progression, but also plays a crucial role in determining the metastatic destination. It is interesting to understand whether small EVs may contribute through their cargo to “prepare” the metastatic site with pro-inflammatory molecules or growth factors that modify vascular permeability and cell recruitment.

## Role of small EVs in the cross-talk between tumor and stromal cells in the TME

### Small EVs in the cross-talk between tumor and immune cells

In the animated, ever-evolving ecosystem of TME, small EVs are the silent messengers that facilitate the intricate and often covert communication between cancer cells and their surrounding neighbors, orchestrating a cellular dialogue that is pivotal in shaping tumor progression in BC. Small EVs released by BC cells play a key role in shaping the immune profile of TME, enabling immune evasion and promoting a pro-tumor immune environment. These vesicles, and particularly their contents, may be able to modulate the activity and phenotype of different immune cell populations. Several studies in BC have looked at the role of small EVs in this cross-talk [45].

Small EVs influence lymphocytes by modulating their proliferation, activation, metabolism, and differentiation, thereby impacting immune responses and disease processes [46]. BC small EVs are involved in immune evasion through direct interference with the anti-tumor immune response.

#### T lymphocytes

Tumor-derived small EVs are known to induce the activation and functional alteration of T lymphocytes. Recent studies have examined the effect of cancer-derived small EVs on the glucose metabolism of cytotoxic CD8 + T cells. In detail, small EVs were isolated from MDA-MB-231 TNBC and from luminal MCF-7 and T47D cell lines and co-cultured with CD8 + T cells isolated from PBMC of healthy females. These vesicles were shown to reduce glucose metabolism in T cells by down-regulating the AKT/mTOR pathway. Additionally, 4T1 tumor bearing mice treated with GW4869, an *exosome* release blocker, showed reduced tumor burden, likely due to an increase in tumor-infiltrating CD8 + T cells in treated mice compared to controls [47].

The alteration of CD8 + T cell functionality is one of the main reasons for the immune escape of TNBC. It has been shown that circulating miR-20a-5p, released by TNBC cells via small EVs, might induce CD8 + T cell dysfunction and contribute to immunotherapy resistance [48]. This study suggests that targeting cirmiR-20a-5p could be a novel strategy to restore TNBC response to therapy. Interestingly, BC cells can also release small EVs that carry immunosuppressive molecules, such as PD-L1, which binds to the PD-1 receptor on T cells, leading to T cell exhaustion and reduced anti-tumor immunity [49]. Moreover, *Chatterjee et al.* demonstrated that changes in the levels of transforming growth factor beta (TGF-β) coordinate PD-L1 + small EVs secretion from BC cells. Cooperative effects of TGF-β and tumor-derived small EVs expressing high PD-L1 impair CD8 + T cell function by suppressing T cell receptor (TCR) signaling mediated phosphorylation [50]. In a recent publication, CD4 + T cells were, instead, the focus of the investigation. Indeed, Chaperonin-containing TCP1 subunit 2 (CCT2), which high levels were found significantly associated to adverse outcome in BC patients, can be delivered, through small EVs, to CD4 + T cells and suppress their activation via CD40L downmodulation [51]. Thus, targeting tumor-derived small EVs and their immunosuppressive cargo, such as cirmiR-20a-5p and PD-L1, presents a promising strategy to restore CD8 + and CD4 + T cell functionality and enhance anti-tumor immunity in TNBC (Table 1a).

#### Macrophages

Macrophages are essential immune cells involved in antigen presentation, phagocytosis, and immunomodulation. Their functional phenotypes are versatile and depend on the tissue type and the signals presented in its microenvironment; they can play multiple roles in the inflammatory process [52]. Numerous studies have demonstrated the interaction between small EVs and macrophages in BC, as tumor-associated macrophages (TAMs) are key components of TME. Central to this is the effect small EVs have on macrophages, highly plastic immune cells that can differentiate into two main functional phenotypes: M1 macrophages, which exhibit pro-inflammatory activity and anti-tumor properties, and M2 macrophages, which play an immunosuppressive role by promoting tumor progression [53]. Small EVs released from BC cells have been shown to induce macrophage polarization towards the M2 phenotype by carrying specific molecular components [54]. Recent studies in 2024 have highlighted the importance of small EVs in modulating this process, particularly in reinforcing the two-way communication between BC cells and macrophages. One study demonstrated the role of exosomal-miR-191-5p, released from BC cells, in favoring the polarization to the M2 phenotype. This miRNA acts by inhibiting suppressor of cytokine signaling 3 (SOCS3) in macrophages, thereby increasing their pro-tumor activity and enhancing tumor growth and metastasis. M2-polarized macrophages, in turn, further promote tumor invasiveness, creating a feedback loop [55]. In TNBC, *Qiao et al.*. have shown that Ras-related protein RAB5A affects the exosome-mediated polarization of macrophages towards the M2 phenotype. The researchers found that RAB5A depletion in TNBC cells significantly reduced exosome secretion and blocked the ability to induce macrophage M2 polarization, a process known to promote tumor progression. Through an analysis of miRNAs associated with macrophage polarization, miR-21 was identified as a key component in tumor cell-derived small EVs. The increased expression of miR-21 in macrophages, induced by tumor cell small EVs, enhanced the bias towards the M2 phenotype. This phenomenon was further elucidated by identifying Pellino-1 (PELI1) as a direct target of miR-21. The overexpression of PELI1 in macrophages partially counteracted the effect of miR-21-induced M2 polarization, suggesting a critical regulatory mechanism. In vivo experiments with xenograft models were performed, and RAB5A knockdown TNBC cells formed smaller and less aggressive tumors, with reduced recruitment of tumor-associated macrophages [56]. In their work, *Mao et al.;* analyzed differential miRNAs in the small EVs of paclitaxel-resistant and responsive BC cell lines using miRNA-seq. It was shown that drug-resistant BC cells and their small EVs expressed more miR-99b-3p than the sensitive ones and their small EVs. miR-99b-3p acted by targeting PPP2CA–protein phosphatase 2 catalytic subunit alpha to promote AKT/mTOR phosphorylation. Drug-resistance was determined also by the presence of M2 polarized macrophages due to miR-99b-3p internalization. Downregulation of miR-99b-3p in vivo reversed M2 polarization of macrophages, suppresses tumor development, and prevented treatment resistance [57]. In another study, *Ma D* and colleagues investigated the mechanism by which M2 macrophage-derived small EVs promoted the development of BC. They found out that co-culturing M2 macrophage-derived small EVs with BC tumor cells determined an increase in lncRNA LINC00470, myc, and DNA (cytosine-5)-methyltransferase 3 A (DNMT3A) expression levels and a downmodulation of miR-199a-3p, resulting in a higher proliferation rate and invasive ability of tumor cells. Moreover, either the inhibition of LINC00470 in the tumor or macrophage small EVs, or the overexpression of miR-199a-3p were able to revert the biological effect reported [58]. In addition to miRNA, tumor-derived small EVs can carry circular RNAs (circRNAs) that influence the behavior of immune cells. A recent study identified *exosomal* circSERPINE2 as a potent regulator of TAMs, promoting their IL-6_secreting phenotype through the MALT1-NF-kB signaling axis, which in turn enhanced BC progression. Silencing this circRNA using a nanoparticle-based delivery system effectively suppressed tumor growth in vivo, suggesting its potential as a therapeutic target to disrupt tumor-immune cell communication [59]. Taking all together, targeting tumor-derived small EVs components, such as miRNAs and circRNAs, offers a promising strategy to inhibit macrophage M2 polarization, disrupt tumor-immune cell interactions, and overcome therapy resistance in BC (Table 1a).

#### Myeloid-derived suppressor cells (MDSCs)

MDSCs are a subset of heterogeneous immature myeloid cells that play a pivotal role in shaping a TME not permissive for an efficient activity of the immune system. It has been shown that this immunosuppressive phenotype can be promoted by BC cells, for instance, through the delivery via small EVs of miR-9 and miR-181a to early myeloid cells. The two miRNAs, by inhibiting SOCS3 and PIAS3, negative regulators of JAK/STAT signaling pathway, triggered MDSC expansion and T-cell immunity suppression [60]. *Liu Q.W.* and colleagues described that BC-released small EVs can downregulate the expression of chemokine receptor CXCR4 and increase the levels of inflammatory cytokines IL-6 and IL-10 in myeloid cells, thus impairing T cell proliferation; MDSC expansion was further stimulated through the activation of STAT3 signaling pathway [61].

An interesting study by *Deshane JS’s* lab evaluated the impact of mechanical forces within the TME on BC progression. It is already well known that a stiff extracellular matrix favors tumor and stromal cell migration and invasion, but what pertains to the topic of this review is that exposure to an oscillatory strain induced an increase in small EVs production, particularly of PD-L1 + small EVs, by TNBC cells, concomitantly to a rise in the number of MDSCs and M2 macrophages, thus resulting in a highly aggressive disease supported by an immunosuppressing TME [26, 62]. The reprogramming of myeloid cells to a pro-tumoral phenotype is reportedly crucial also at the metastatic site, where these cells favor circulating tumor cell homing to the secondary organ as will be detailed in Sect. 5. In conclusion, BC-derived small EVs drive MDSC expansion and immune suppression through, for example, miRNA-mediated JAK/STAT activation and inflammatory cytokine modulation, highlighting their role in fostering an immunosuppressive and metastatic TME (Table 1a).

### Small EVs in the cross-talk between tumor and non-immune stromal cells

#### Fibroblasts

Being the principal cellular component of the TME, fibroblasts and their direct cross-talk with the other neighboring cells, have been extensively studied through the years, especially in BC. In 2016, our group demonstrated that the metastamir miR-9 is released by TNBC cells and delivered, packaged into small EVs, to the surrounding fibroblasts to induce a CAF-like phenotype in turn leading to an increase in tumor aggressiveness [63]. This is a *modus operandi* that, nowadays, is well recognized and has been described in all BC subtypes. For example, in the context of Luminal A tumors, *Ahn S.* and colleagues demonstrated that BC cells transfer miR-130b-3p to CAFs to activate them by targeting the nucleation-promoting factor adaptor protein SPIN90 [64]. CAFs also release small EVs to affect the behavior of the tumor and the other stromal cells. In the study by *Wu H.J. et al.*, the authors showed that the expression of the oncogene focal adhesion kinase (FAK) in CAFs controls miRNA content in small EVs and, consequently, the migratory capability of the recipient tumor cells [65]. Another work from the group of *Babashah S.*, instead, illustrated that CAF-educated monocytes differentiate into pro-tumoral M2 macrophages, which are able to transfer, through small EVs, miR-181a to inhibit the onco-suppressor PTEN and activate a survival signaling cascade in BC cells [66]. As already mentioned, this aberrant tumor-CAF cross-talk is also able to affect response to therapy. It was recently discovered through single cell RNA sequencing of ER + BCs that a specific subset of CAFs (CD36 + CAFs) was highly enriched in tumor tissues resistant to Cyclin-dependent kinase (CDK) 4 and 6 inhibitors (CDK4/6i) [67]. A more detailed analysis revealed that the resistant phenotype was induced by CD36 + CAFs through the release of miR-20-containing small EVs, resulting in the downregulation of the tumor-suppressor retinoblastoma 1 (RB1) in BC cells. In conclusion, tumors and *corrupted* fibroblasts are powerful *partners in crime* in BC TME and small EVs are the malleable bullets exploited to favor tumor survival and progression (Table 1b).

#### Endothelial cells

Angiogenesis is a crucial step in cancer progression. Indeed, also endothelial cells are pushed by the craving tumor to activate, proliferate, migrate and form new vasculature for blood supply. MicroRNAs are often the most studied small EVs cargo but lncRNAs and proteins can function as aberrant messages too. *Liu J et al.;* described the role of the lncRNA Metastasis Associated Lung Adenocarcinoma Transcript 1 (MALAT1) in the promotion of angiogenesis in TNBC TME: in Jagged-1 (JAG1, ligand of NOTCH signaling pathway) overexpressing BC cells the release of small EVs is promoted and MALAT1 is transferred to endothelial cells, where it targets miR-140-5p [68]. The tyrosine kinase EphrinA2 (EphA2) has been implicated in different oncogenic processes, including angiogenesis. In 2022, it was demonstrated that exosomal EphA2 from TNBC cells was able to increase endothelial cell migration and vascular permeability, thus favoring tumor metastatization [69]. Moreover, drug-resistant tumor cell-derived small EVs have also been found to be enriched in EphA2 and linked to the induction of a higher invasive capability of recipient sensitive BC cells [70]. Hypoxia is a prime stimulus of angiogenesis. In this context, *Ning W.*,* Yang J.*,* Ni R.* and colleagues have recently shown that LncRNA Ribonuclease P RNA component H1 (RPPH1) loading into small EVs was facilitated under hypoxic conditions, and its internalization in both BC and endothelial cells promoted the activation of the pro-tumoral PI3K/AKT signaling pathway [71]. In conclusion, angiogenesis plays a pivotal role in cancer progression, with tumor-derived small EVs, and their heterogeneous content, serving as key mediators of communication between BC cells and endothelial cells to drive vascular development (Table 1b).

#### Adipocytes

The adipose tissue is a major component of the mammary gland and its plasticity is a key feature required in organ maturation and during pregnancy, even though the whole process is yet to be thoroughly described. Nevertheless, what is increasingly evident is that adipose cells, in all their stages of differentiation, and their cross-talk with tumor cells impact cancer evolution [72]. For example, *Qin Zhu* and colleagues have characterized the effects of adipose-derived stem cells (ASCs) and their small EVs (ASC-exos) on T cells, macrophages and tumor cells in BC. The authors discovered that ASC-exos induce the acquisition of an immunosuppressive phenotype in both immune cells and a more aggressive behavior in cancer cells [73].

Exosome content can mirror racial disparities in disease outcomes. Indeed, the interesting work by *Zhao D et al.;* identified miR-1304-3p as the most upregulated miRNA in African American compared to Caucasian American patients, and linked it to a poorer prognosis. This difference was caused by a single nucleotide polymorphism in the stem-loop region of the miRNA. Moreover, exosomal miR-1304-3p, through the targeting of GATA2, a transcription factor involved in stem cell maintenance, was seen to activate cancer-associated adipocytes and induce the release of lipids, fueling cancer cell growth [74].

Finally, the adipose tissue may also have a protective role against tumor survival and progression. *Wang X.’s* group investigated the impact of adipose-derived small EVs on response to therapy and found out that ASC-exos carrying miR-1236 increased response to cisplatin in BC cells by suppressing Solute Carrier Family 9 Member A1 (SLC9A1), an isoform of sodium–hydrogen antiporter, and the Wnt/β-Catenin Signaling [75]. In summary, the multifaceted role of adipose tissue and its small EVs in BC highlights its dual nature and understanding these dynamics provides critical insights into potential therapeutic strategies (Table 1b).

### Physicochemical properties of the TME regulation of small EVs cargo in BC

The TME plays a critical role in modulating small EVs composition and function in BC, with factors such as stiffness, hypoxia, and acidification significantly influencing the molecular cargo of these vesicles. Several studies suggest that altering the mechano-transduction pathways leads to the selective enrichment of specific proteins and miRNAs cargo Small EVs, involved in tumor progression, EMT, and therapy resistance. For example *exosomal* small nucleolar host gene 1 (SNHG1) from hypoxic BC cells promote tumor angiogenesis and growth via regulating miR-216b-5p/JAK2 axis [76]. One of the most consistently upregulated microRNAs in hypoxic tumors is miR-210, often referred to as a “master hypoxamir.” In BC, miR-210 facilitates tumor adaptation to hypoxic stress by promoting angiogenesis, cell survival, migration, and radiotherapy resistance. Its elevated levels are associated with poor prognosis and enhanced metastatic potential, making it a candidate biomarker for hypoxic burden and therapeutic response in BC patients [77]. Small EVs also induce ECM remodelling, and conversely, biophysical cues encoded by ECM may also influence small EVs biogenesis and cargo sorting. One study demonstrated the instructive role of ECM stiffness in regulating *exosome* secretion and its effects on BC cell motility and migration, and identifies *exosomal* thrombospondin 1 (THBS1) as the master regulator of cancer invasion [78]. Thus, the TME dynamically shapes the composition of small EVs in BC, with factors like hypoxia and ECM stiffness influencing vesicle cargo to promote angiogenesis, metastasis, and therapy resistance, highlighting potential targets for therapeutic intervention (Table 1c).

## Contribute of small EVs and their cargo to BC metastatization

One of the most interesting aspects of small EVs in the context of BC is their involvement in metastatic organotropism, the tendency of cancer cells to form metastases in specific organs such as brain, bone, lung and liver [79]. This phenomenon is mediated by a complex molecular dialogue played out also by small EVs [80]. Small EVs can serve as vehicles for horizontal transfer of genetic material and proteins, promoting modifications in the TME and allowing a tumor permissive soil in distant tissues. Their contribution to metastatic organotropism occurs through the preparation of the pre-metastatic niche: small EVs derived from the primary tumor can *‘educate’* stromal cells in the target organs both by promoting a favorable environment for metastatic cells to take root and through the expression of surface proteins, including integrins, which direct the uptake of small EVs by specific target tissues. For instance, the important work by *Hoshino A*. and colleagues demonstrated that integrins α6β4 and α6β1 are associated with lung tropism, whereas integrin αvβ5 is linked to liver metastasis. BC is known to metastasize to specific organs, particularly brain, bones, lung, liver and distant lymph nodes [81]. Here we discuss the latest studies describing the role played by small EVs in conveying information from the primary tumor to premetastatic sites and the modification of the microenvironment at the “target” district.

### Metastatization to the brain

Brain metastasis significantly contributes to the elevated mortality in BC patients. In 2023, *Sakamoto et al.* summarized the molecular mechanisms by which small EVs are involved in BC brain metastasis [82]. The work by *Lu Y et al.;* explores the role of small EVs in the integrity of the blood-brain barrier (BBB) [83]. In particular, the authors firstly established a highly brain metastatic BC cell line, analyzed the content of the released small EVs, and discovered high levels of the long non-coding RNA GS1-600G8.5. The internalization by brain microvascular endothelial cells of these small EVs resulted in an increased permeability of the BBB to the crossing of BC cells.

### Metastatization to the bone

Bone metastasis is a characteristic of BC, particularly ER + malignancies, and is associated with extensive bone remodeling. In 2021, the group of *Ravi Singh and Kounosuke Watabe* found increased levels of Integrin-Binding Sialoprotein (IBSP) and miR-19a in the small EVs of bone-tropic ER + BC cells [84]. Interestingly, IBSP attracts osteoclasts to the bone TME and assists miR-19a internalization, thus promoting osteoclastogenesis and the creation of a permissive environment for tumor colonization.

Another recent study investigated how *exosomal* ICAM1 helps TNBC cells to spread to the bone. Researchers found that TNBC cells release ICAM1-enriched small EVs and the binding of ICAM1 to its receptor is necessary for the suppressive effect on CD8 + T cell activity, creating an immunosuppressive TME that may contribute to TNBC tumor growth and bone metastasis. Blocking intercellular adhesion molecule 1 (ICAM1) significantly impairs the ability of small EVs released by BC to bind to CD8 + T cells, thereby inhibiting their immunosuppressive effects [85].

### Metastatization to the lungs

If luminal BCs preferentially metastasize to the bone, basal-like tumors show a lung tropism. A study by *Qi et al.;* showed that TNBCs express high levels of the evolutionarily conserved RNA-binding protein Lin28B, which induce an enrichment in epithelial ALDH + BC stem cells. These cells are the primary source of small EVs with low levels of the tumor-suppressor miRNA let-7s. The release of these small EVs in the lung pre-metastatic niche recruits and converts neutrophils to immunosuppressive N2 cells, thus supporting cancer progression [86]. Another study investigated the role of small EVs in the transport of the membrane protein Caveolin-1 (Cav-1) between primary BC and the microenvironment of metastatic sites [87]. It was demonstrated that Cav-1 expressing small EVs prepare a suitable metastatic niche for BC cell homing by regulating: (1) the expression of PMN marker genes and inflammatory chemokines in lung epithelial cells, (2) the deposition of ECM matrix by lung fibroblasts, and (3) the M2-type polarization of resident macrophages. Understanding the interplay between microvesicle cargo and organotropism offers new opportunities for therapeutic interventions. Blocking the release or uptake of specific small EVs, or targeting their cargo, could potentially disrupt the metastatic cascade and improve patient outcomes (Table 1d).

## Small EVs released from immune and stromal cells in tumor progression

Small EVs released by tumor cells (TDEs) play a crucial role in shaping the tumor microenvironment and influencing recipient cells by transferring oncogenic signals, promoting immune evasion, and facilitating metastasis. However, small EVs secreted by resident stromal and immune cells within the microenvironment also exert distinct functional effects. These vesicles can act in an autocrine manner, influencing the same cell type that released them; in a heterologous manner, affecting different cell populations, including tumor cells; or in a paralogous manner, mediating communication between similar but distinct cell lineages. This bidirectional exchange of small EVs’ cargo between tumor and resident cells contributes to tumor progression, therapy resistance, and microenvironmental remodelling. While TDEs have been extensively studied in the context of tumor progression, small EVs from immune and stromal cells also play critical roles in cancer invasiveness and progression. These small EVs contribute to tumor modulation, therapy resistance, and immune response reprogramming, thereby influencing disease outcomes [88].

### Immune cell-derived small EVs

Macrophage-derived small EVs have been shown to exert immunomodulatory effects in the TME. For instance, M2 macrophage-derived small EVs carry miR-21, which enhances tumor cell invasion and metastasis [89]. *Exosomal* miR-223 from macrophages can also be delivered to BC cells, which can result in myocyte enhancer factor 2c (Mef2c) suppression and therefore lead to invasion and metastasis [90]. *Chen et al.*. have found that macrophage-derived small EVs delivered lncRNA HISLA into BC cells and then regulated cancer cell glycolysis by combining with prolyl hydroxylase domain 2 (PHD2) to maintain HIF-1α stable [91], indicating that macrophage-derived *exosomal* lncRNAs have a positive role in tumor progression. In another study, M2 macrophages switch into the M1 phenotype through direct and/or indirect ways, and then their small EVs resurgent cancer cells by activating NF-кB pathway in BC with bone metastasis [92]. When macrophages were exposed to apoptotic BC cells after chemotherapy, they increased the secretion of IL-6 in small EVs and then delivered it to cancer cells to promote proliferation and metastasis through increased STAT3 phosphorylation [93]. Additionally, dendritic cell-derived small EVs can transfer tumor-associated antigens to T cells, influencing anti-tumor immunity [94].

### Stromal cell-derived small EVs

Fibroblast-derived small EVs contribute to ECM remodelling and cancer progression. CAF-derived *exosomal* miR-181a suppresses tumor suppressors, such as PTEN, thereby enhancing BC cell survival and invasion [66]. MSC-derived *exosome* incorporation by BC cell line MCF7 stimulates growth and migration and protects against serum starvation and chemotherapeutic drugs in vitro. In a mouse xenograft model, exosome depletion reduces the tumor-promoting effects of adipocytes. Transcriptomic analysis of MSC-derived exosome-treated MCF7 identifies several signaling cascades, including the Hippo pathway. Blockade of this pathway reduces the growth-promoting effect of adipocyte exosome signaling [95]. Similarly, small EVs from endothelial cells facilitate angiogenesis by delivering pro-angiogenic molecules such as VEGF and lncRNA MALAT1 to tumor cells [96, 97] (Table 1e).

**Table 1:** Literature schematic summary

a. Small EVs in the cross-talk between tumor and immune cells			
Immune Cell Type	Small EVs Cargo	Effect on Tumor	Reference
**T Lymphocytes**	miR-20a-5p, PD-L1	Immune evasion by suppressing CD8 + T cells	[48]
**Macrophages**	miR-191-5p, miR-21	Promotes M2 macrophage polarization, supports tumor growth	[55, 56]
**Myeloid-derived Suppressor Cells (MDSCs)**	miR-9, miR-181a	Suppresses T cell proliferation, increases immune suppression	[60]
**b. Small EVs in the cross-talk between tumor and non-immune stromal cell**			
***Stromal Cell Type***	***Small EVs Cargo***	***Effect on Tumor***	***Reference***
**Fibroblasts**	miR-9, miR-181a	Induces CAF-like phenotype enhances tumor aggressiveness	[66–95]
**Endothelial Cells**	lncRNA MALAT1, RPPH1	Promotes angiogenesis, enhances tumor metastasis	[68, 71]
**Adipocytes**	miR-1304-3p	Promotes tumor metabolism and progression	[74]
**c. Physicochemical Properties of the TME Regulation of small EVs Cargo in BC**			
***Physicochemical Property***	***Small EVs Cargo Affected***	***Effect on Tumor Progression***	***Reference***
**Hypoxia**	SNHG1, miR-216b-5p;	Promotes angiogenesis; tumor growth	[76]
**Stiffness**	THBS1	Regulates cancer invasion, enhances motility	[78]
**d. Contribution of small EVs cargo to BC metastatization**			
***Metastatic Organ***	***Small EVs Cargo***	***Effect on Pre-Metastatic Niche***	***Reference***
**Brain**	GS1-600G8.5	Increases blood-brain barrier permeability for tumor invasion	[81]
**Bone**	IBSP, miR-19a	Promotes osteoclastogenesis, facilitates bone metastasis	[82, 83]
**Lung**	Caveolin-1	Recruits neutrophils modulates lung microenvironment	[85]
**e. Small EVs released from Immune and Stromal Cells in Tumor Progression**			
***Cell Type***	***Small EVs Cargo***	***Effect on Tumor***	***Reference***
**Macrophages**	miR-21, miR-223, lncRNA HISLA	Supports immune suppression, enhances tumor growth	[87, 88, 89]
**Fibroblasts**	miR-181a	Increases tumor invasion and therapy resistance,	[95]

## Small EVs as a tool for precision oncology in BC

**Fig. 2:**
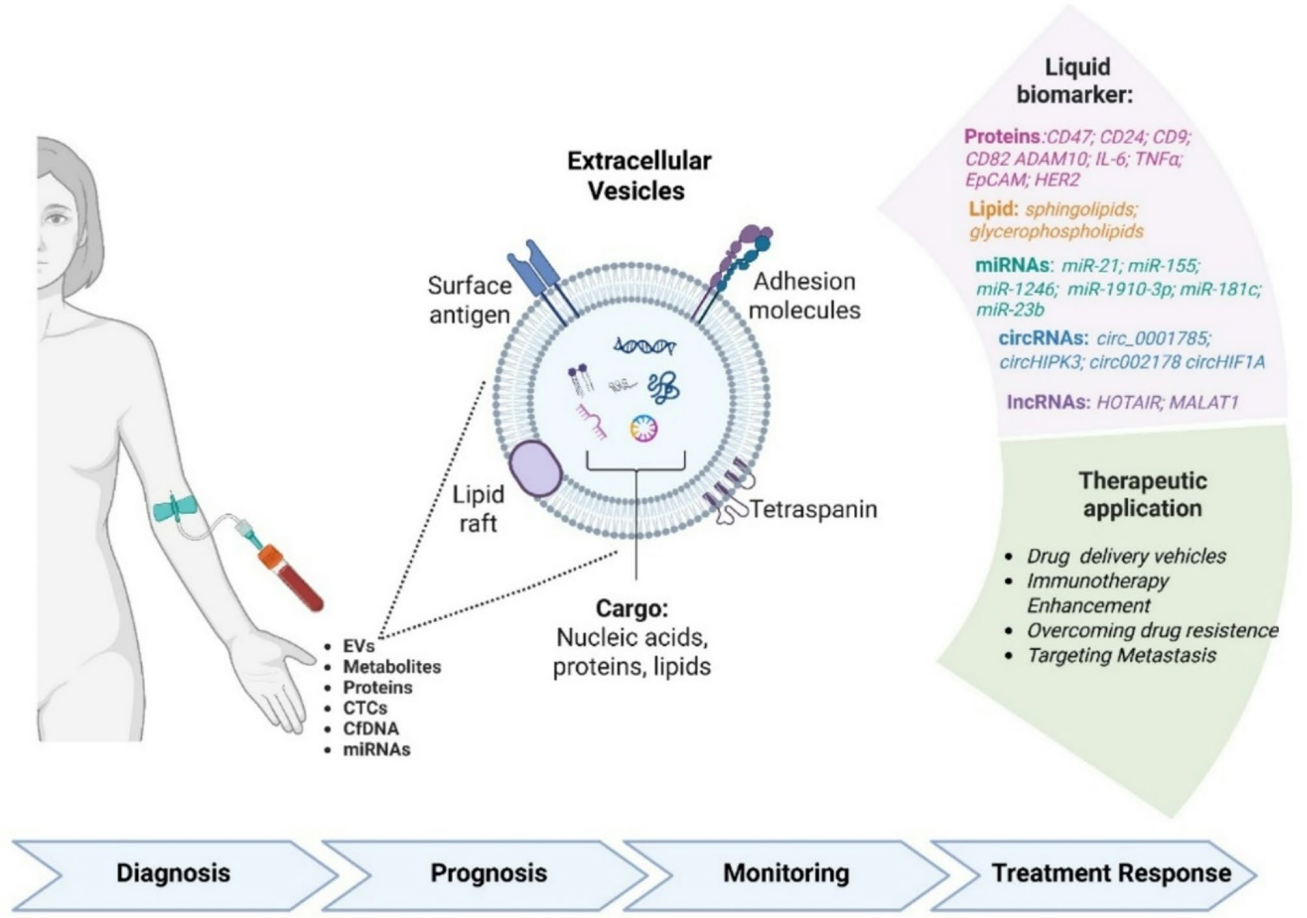
Extracellular vesicles (EVs) as liquid biopsy tools and therapeutic vehicles in BC. The panel illustrates the structural and functional features of EVs, highlighting their diverse cargo—nucleic acids, proteins, and lipids—and surface components including tetraspanins, adhesion molecules, and antigens. EVs can be isolated from patient blood samples along with other liquid biopsy components such as circulating tumor cells (CTCs), circulating free DNA (cfDNA), proteins, metabolites, and microRNAs (miRNAs). Tumor-derived EVs carry bioactive molecules, including specific proteins (e.g., CD47, CD24, CD9, EpCAM), lipids, and non-coding RNAs (miRNAs, circRNAs, lncRNAs), which can serve as diagnostic and prognostic biomarkers. Additionally, EVs hold promise as therapeutic tools in oncology, acting as drug delivery vehicles, immunotherapy enhancers, and agents for overcoming drug resistance or targeting metastasis. EVs represent versatile components of liquid biopsy with potential applications in early diagnosis, prognostic assessment, longitudinal disease monitoring, and evaluation of therapeutic efficacy (Created with BioRender.com)

Small EVs are becoming a groundbreaking tool in precision oncology, with significant potential to enhance BC management through liquid biopsy. These small EVs are released by both healthy and cancerous cells into bodily fluids like blood, urine, and saliva. Tumor-secreted small EVs carry a rich cargo of nucleic acids, proteins, and lipids protected by a lipid bilayer, their contents mirror the molecular characteristics of the originating cells. This stability and tumor specificity make small EVs attractive biomarkers. In fact, these vesicles serve as an important source of biomarkers for diagnosing cancer, predicting outcomes, and monitoring treatment effectiveness (Fig. 2) [98]. Their ability to circulate and reflect tumor evolution makes them a powerful tool for longitudinal monitoring and early diagnosis. Unlike conventional tissue biopsies, liquid biopsies based on small EVs are less invasive and allow repeated sampling to track tumor dynamics in real time [99].

### Isolation and characterization: technical background and challenges

A critical step in utilizing small EVs for liquid biopsy is isolating them from biofluids with sufficient yield and purity. Differential ultracentrifugation (dUC) is the classical “gold standard” method, using sequential high-speed spins to pellet EVs. UC is simple and suitable for large volumes at low cost, often yielding high-purity vesicles. However, UC is labor-intensive and can co-pellet contaminants (protein aggregates, lipoproteins) if not carefully controlled. It also suffers from low particle recovery in small samples and potential vesicle damage under strong centrifugal forces. Density gradient UC (floating EVs in a sucrose or iodixanol gradient) further improves purity by separating vesicles from contaminants of similar size/density, but adds complexity and time [100]. Size-exclusion chromatography provides a gentle alternative by eluting particles based on hydrodynamic radius. It effectively preserves vesicle integrity and reduces protein contamination, but fails to discriminate between EVs and other similarly sized nanoparticles such as lipoproteins. Immunoaffinity-based isolation employs antibodies targeting EV surface markers (e.g., CD63, CD81), enabling enrichment of specific subpopulations. While highly specific, these approaches are often limited by cost, scalability, and variability in antigen expression across EV sources. Microfluidic devices integrate both physical (e.g., size-based filtration, acoustic separation) and affinity-based mechanisms into miniaturized platforms, offering rapid and high-throughput EV isolation from small sample volumes. Nonetheless, these systems require complex fabrication and lack standardization, which hinders their clinical adoption [33]. Small EVs’ characterization typically involves assessing their size (30–200 nm) and identifying specific markers, such as CD9, CD63, and CD81, through techniques like Western blot or flow cytometry. Proteins like HSP70, Alix, and TSG101, associated with EV formation and function, are also commonly analyzed. Advanced methods, including scanning electron microscopy, transmission electron microscopy, and nanoparticle tracking analysis, help determine morphology and concentration [101].

### Diagnostic applications

Once isolated, small EVs reveal a wealth of *disease-specific* information. Several studies exploit the various omics sciences, such as proteomics, genomics, and transcriptomics, to analyze microvesicle content. Since most BC-related deaths result from metastatic disease, the clinical importance of small EV research largely lies in their role in establishing pre-metastatic niches and facilitating tumor cell spread. This makes small EVs valuable biomarkers for predicting treatment response and metastasis. Liquid biopsies rely on the detection of circulating small EVs as biomarkers for early cancer diagnosis and monitoring. For example, *exosomal* miR-21 [102] has recently shown subtype-dependent diagnostic potential, particularly correlating with tumor aggressiveness and proliferation in HER2 + and triple-negative cases [103]. Other miRNAs such as, miR-155, miR-1246, 1910-3p [104], miR-200b-3p, miR-181c have been identified as potential diagnostic biomarkers for BC, correlating with tumor progression and metastasis [105]. Several circular RNAs encapsulated in EVs, including *circ_0001785*,* circ_002178*, and *circHIPK3*, have been identified in BC patients, they are involved in tumor progression and they are used as biomarkers for cancer detection and prognosis [106]. Their stability in body fluids and specific expression patterns make them promising candidates for liquid biopsy applications [107]. In addition to nucleic acids, recent evidence highlights the diagnostic value of EVs-associated lipids in liquid biopsy; notably, a distinct panel of sphingolipids and glycerophospholipids has been shown to effectively distinguish BC patients from healthy controls, reinforcing the role of EV lipidomics in early detection strategies [108]. EVs also carry tumor-specific proteins that can serve as biomarkers for BC detection and subtyping. These include CD9, ADAM10, CD82+, CD24, CD47 and EpCAM, their expression increases significantly with the progression of malignant BC [105]. Additionally, *exosomal* PD-L1 levels in patient plasma have been proposed as a predictive biomarker for immunotherapy response. Besides their diagnostic and prognostic values, small EVs may also modulate susceptibility to HER2-targeted therapy. Indeed, it has been reported that HER2-overexpressing small EVs may contribute to decreasing trastuzumab benefit by acting as molecular decoys able to sequester the biodrug or transfer HER2-driven pro-oncogenic signaling to recipient cells, thereby diminishing the effectiveness of targeted therapy [109]. Recent proteomic profiling of serum-derived EVs has identified diagnostic signatures and potential therapeutic targets in BC, including TALDO1, a protein linked to metastatic progression and now being explored for targeted inhibition [110]. However, despite these advantages, *exosome-based* biomarkers have not yet reached the level of diagnostic accuracy, validation, and regulatory acceptance of current clinical standards such as mammography and tissue biopsy. Mammography remains the frontline screening tool for BC, supported by large-scale population data and established clinical guidelines, despite limitations in sensitivity, especially in women with dense breast tissue [111, 112]. Tissue biopsy, while invasive, continues to provide definitive histopathological and molecular information that forms the basis for therapeutic decisions. At present, liquid biopsy using EVs should be regarded as a complementary tool—particularly valuable for longitudinal monitoring, inaccessible tumors, or when repeated tissue sampling is unfeasible—rather than a full substitute for conventional diagnostic procedures [113]. Several clinical trials have been initiated to test the diagnostic potential of small EVs in BC. Table 2 summarizes different clinical trials evaluating the use of small EVs in BC patients (ClinicalTrials.gov). These studies explore the potential of EVs as biomarkers and therapeutic targets [114]. Nevertheless, key limitations persist. Small EV heterogeneity, both intra- and inter-patient, complicates biomarker discovery and interpretation. Standardized protocols and validated biomarker panels are urgently needed to facilitate clinical translation and regulatory approval.

**Table 2:** Clinical trial: (exosomes and small EVs in BC)

NCT Number	Study Title	Status	Sample Source/ Tumor Type	Study Type
NCT01344109	*A Pilot Study of Tumor-Derived Exosomes as Diagnostic and Prognostic Markers in Breast Cancer Patients Receiving Neoadjuvant Chemotherapy*	WITHDRAWN	N/A N/A	OBSERVATIONAL
NCT05955521	*Exosome as the Prognostic and Predictive Biomarker in EBC Patients*	ACTIVE NOT RECRUITING	N/A TNBC; HER2-positive	INTERVENTIONAL
NCT05286684	*Feasibility of Exosome Analysis in Cerebrospinal Fluid During the Diagnostic Workup of Metastatic Meningitis (Exo-LCR)*	COMPLETED	CSF N/A	INTERVENTIONAL
NCT04653740	*Omic Technologies to Track Resistance to Palbociclib in Metastatic Breast Cancer*	ACTIVE NOT RECRUITING	N/A Advanced BC	INTERVENTIONAL
NCT04530890	*Interest of Circulating Tumor DNA in Digestive and Gynecologic/Breast Cancer*	RECRUITING	Blood N/A	INTERVENTIONAL
NCT04258735	*Genetic Characteristics of Metastatic Breast Cancer Patients*	N/A	N/A Metastatic BC	INTERVENTIONAL
NCT03974204	*Analyses of Exosomes in the Cerebrospinal Fluid for Breast Cancer Patients With Suspicion of Leptomeningeal Metastasis.*	WITHDRAWN	CSF and Blood N/A	INTERVENTIONAL
NCT02977468	*Effects of MK-3475 (Pembrolizumab) on the Breast Tumor Microenvironment in Triple Negative Breast Cancer*	RECRUITING	N/A TNBC	INTERVENTIONAL
NCT04781062	*Development of a Horizontal Data Integration Classifier for Noninvasive Early Diagnosis of Breast Cancer*	N/A	Blood and urine N/A	INTERVENTIONAL
NCT02662621	*Pilot Study With the Aim to Quantify a Stress Protein in the Blood and in the Urine for the Monitoring and Early Diagnosis of Malignant Solid Tumors*	COMPLETED	Blood and Urine N/A	INTERVENTIONAL
NCT04288141	*A Study to Measure the Expression of the HER2-HER3 Dimer in Tumour and Blood (Exosomes) Samples From Patients With HER2 Positive Breast Cancer Receiving HER2 Targeted Therapies*	N/A	Blood and tumor tissue (biopsies); HER2-positive	OBSERVATIONAL
NCT02892734	*Ipilimumab and Nivolumab in Treating Patients With Recurrent Stage IV HER2 Negative Inflammatory Breast Cancer*	TERMINATED	N/A HER2/Neu Negative; Recurrent Inflammatory BC; Stage IV BC	INTERVENTIONAL
NCT04298398	*Impact of Group Psychological Interventions on Extracellular Vesicles in People Who Had Cancer*	N/A	N/A N/A	INTERVENTIONAL
NCT06522971	*Effects of Physical Exercise on Response to Treatement in Breast Cancer*	ACTIVE NOT RECRUITING	N/A N/A	INTERVENTIONAL
NCT03783364	*Pre- or Postoperative Accelerated Radiotherapy*	COMPLETED	N/A N/A	INTERVENTIONAL

### Therapeutic approaches and preclinical evidence

In addition to diagnostics, small EVs are being explored as drug delivery vehicles in BC. Their endogenous origin and ability to cross biological barriers make them attractive carriers for chemotherapeutics, gene therapies, and immune modulators. Several preclinical studies support this potential [113]. For instance, macrophage-derived small EVs loaded with oxaliplatin have demonstrated tumor-targeting capabilities and modulation of tumor physiology in mouse models. These small EVs were then injected into mice to track tumor progression and physiological changes [115]. In another study, researchers developed a poly(lactic-co-glycolic acid)-coated nanocage combined with macrophage-derived small EVs for targeted chemotherapy of TNBCs. To enhance tumor targeting, the *exosome* surface was modified with a peptide that targets the mesenchymal-epithelial transition factor (c-Met), which is overexpressed in TNBC cells. The results showed that nanoparticles coated with engineered small EV significantly improved cellular uptake and the antitumor effectiveness of doxorubicin. In vivo studies revealed that these nanocarriers demonstrated strong tumor-targeting ability, greater inhibition of tumor growth, and increased tumor cell apoptosis. These findings suggest that this strategy holds great promise as a drug delivery system for TNBC treatment [116]. Another study focused on engineering nano-bombs using small EVs derived from CAR-NK cells to enhance therapy for brain metastases of HER2 + BC. Researchers developed a novel biomimetic nanoplatform combining chimeric CAR-NK small EVs (ExoCARs) with a micelle-based nanobomb. This nanocage, termed ExoCAR/T7@Micelle, was designed to improve anti-tumor efficacy by disrupting the ferroptosis defense mechanism. By modifying the small EVs with a transferrin receptor-binding peptide (T7) and expressing CAR on their surface, the nanoplatform successfully crossed the blood-brain barrier and selectively targeted HER2 + BC cells. The integration of reactive oxygen species (ROS)-enhanced and photodynamic therapy (PDT)-based nanobombs enabled precise, site-specific cargo release. In vivo, the orthotopic HER2 + brain metastasis model demonstrated a strong anti-tumor response, significantly improving survival rates. Histological and blood analysis revealed no noticeable side effects. This study is the first to present an effective HER2 + BC brain metastasis treatment using a CAR-T biomimetic drug delivery system [117]. While this strategy offers an innovative proof-of-concept for targeted delivery of EV-based therapeutics to the brain, their clinical translation is limited by several factors. These include manufacturing scalability, consistency of EVs engineering, and regulatory approval. Nevertheless, this work demonstrates the potential of combining cell-based specificity with EV-mediated delivery to treat otherwise untreatable metastatic disease. In a study by *Paul S.* and colleagues, an exosome-based drug delivery system was developed using doxorubicin-encapsulated small EVs (ExoDS) derived from mature dendritic cells (mDCs). The results demonstrated that ExoDS effectively targeted both BC cells and BC stem cells. Importantly, ExoDS did not induce significant apoptosis in healthy breast cells or peripheral blood mononuclear cells (PBMCs) from healthy individuals and BC patients. ExoDS successfully targeted circulating tumor cells isolated from patient blood samples. In vivo, ExoDS showed comparable effectiveness to free doxorubicin. Additionally, in mouse tumor tissues, ExoDS reduced the expression of cancer stem cell markers, highlighting its potential as a targeted therapeutic approach [118]. Two ongoing clinical trials (NCT05955521; NCT05831397) are currently evaluating the use of EVs in BC patients following neoadjuvant chemotherapy. and a strategy to prevent therapy-insensitive cells transferring resistance profiles to sensitive cells could involve integrating EV inhibitors with chemotherapy regimens [114]. However, the translational trajectory of these innovations remains uncertain. While their in vivo performance seems encouraging, EV’s clinical application is limited by key issues: variable targeting efficiency, challenges in ensuring stability and reproducibility, and a lack of GMP-compliant scalable manufacturing. Immunogenicity and regulatory concerns further complicate their integration into clinical protocols [119]. Thus, while small EV-based therapies hold substantial promise for treating aggressive or metastatic BC—including hard-to-reach sites like brain metastases—the field must address major obstacles to achieve clinical readiness. Further studies are needed to standardize production, validate safety, and quantify long-term therapeutic outcomes in humans.

### Challenges in clinical translation of small evs: heterogeneity and standardization barriers

In the field of precision oncology, the use of small EVs as a diagnostic and therapeutic tool has considerable prospects. However, despite increasing preclinical success, several unresolved challenges hinder their broad clinical implementation. One of the most pressing limitations is the intrinsic heterogeneity of EV populations [98]. Tumor cells release a several vesicles that vary in size, surface markers, and cargo composition, reflecting not only inter-patient variability but also intra-tumoral heterogeneit [120]. This biological diversity represents significant problems for the reproducibility and reliability of liquid biopsy results. For instance, a specific EV biomarker may be present in one patient’s vesicles but absent or underrepresented in another, complicating early diagnosis and treatment stratification [121]. From a therapeutic standpoint, heterogeneity implies that EV preparations are mixtures, where only a subset may carry the desired functional cargo or exhibit appropriate targeting properties. This raises concerns about off-target effects, batch consistency, and potency. Efforts to mitigate these issues include the development of microfluidic and immunoaffinity-based platforms for subtype-specific EV isolation. Sorting EVs by size, density, or specific markers such as CD9 or EpCAM may help enrich for functionally relevant subpopulations [33]. However, these technologies remain expensive, technically demanding, and lack standardization for clinical adoption. Another unresolved issue is the poor understanding of how EV subtypes correlate with functional roles. For example, which molecular signatures distinguish metastasis-promoting vesicles from non-pathogenic ones? Which size or cargo profile is associated with drug resistance transmission? Addressing these questions is crucial for designing truly effective and safe EV-based interventions. Moreover, the lack of robust in vivo tracking methods limits our ability to evaluate EV biodistribution, half-life, and organ-specific accumulation—parameters essential for both therapeutic optimization and regulatory approval [122]. Combined with the absence of consensus protocols and Good Manufacturing Practice (GMP)-compliant workflows, these limitations constitute major bottlenecks to clinical translation. Integrating emerging technologies like single-cell sequencing, spatial transcriptomics, and multi-omics profiling may help overcome current barriers and advance EV-based applications in personalized BC care [123, 124]. The field still needs a deeper understanding of how EV heterogeneity correlates with function, so that therapies can target the “bad actors.”

## Emerging technologies and trends in small EVs research

In BC, the field of small EV research is changing quickly as new technology provide opportunities to better understand and utilize these vehicles. However, the complexity and heterogeneity of small EV populations, combined with the large amount of molecular data they generate, require advanced computational tools to unlock their full diagnostic and therapeutic potential. Artificial intelligence (AI), which is commonly defined as the simulation of human intelligence by machines, and its subdomain, machine learning (ML), which focuses on pattern recognition and predictive modelling using data, are playing an increasingly important role in this field [125]. AI tools such as ML, deep learning (DL), and natural language processing (NLP) are very good at understanding the intricate chemical data found in EVs. Rapid, high-throughput analysis of microvesicles cargo is made possible by these methods, as they also enable the automated detection of disease-specific markers with remarkable sensitivity and specificity [126]. AI methods, such as ML, are becoming more and more important for converting small EVs into clinical insights. Small EVs have become important non-invasive indicators in BC. In the context of BC, AI-driven platforms enable the integration of big data from diverse sources such as blood-based small EV profiles (liquid biopsies), histopathology and radiology images, and genomic analyses, a comprehensive view of tumor biology and bridging information limitations [111]. Notably, AI models can analyze small EV-derived multi-omics datasets to uncover subtle disease patterns, improve early detection accuracy, and predict therapeutic responses [127]. For instance, ML algorithms have identified novel EV-associated molecular signatures and gene hubs linked to BC progression and prognosis [128]. Crucially, these technologies allow accurate mapping of intratumoral heterogeneity at the molecular level by decoding the complex cargo of circulating small EVs, supporting patient stratification and individualized therapy – key goals of precision medicine [126]. In fact, recent reviews underscore that EVs are becoming “new weapons” in precision oncology but require AI-driven analytical support to fully exploit their rich information [128], mirroring the broad impact of AI across the BC care continuum from enhanced screening detection to personalized treatment planning [111, 129].

## Conclusions

Small EVs have been demonstrated to play a key role in BC development, proliferation, and progression by mediating tumor-stroma communication, immune evasion, and metastatic dissemination, thus revealing a broad spectrum of biological functions, including both immune tolerance and stimulation. In addition to tumor-derived small EVs, vesicles secreted by immune and stromal cells also play a significant role in shaping the TME, modulating therapy resistance and sustaining cancer cell survival. Clinically, their potential as biomarkers for liquid biopsy and vehicles for targeted drug delivery is increasingly recognized, particularly in hard-to-reach metastatic settings like the brain. However, the gap between experimental promise and clinical application will need to be bridged by standardized isolation and characterization techniques, in vivo tracking, the validation of EV-associated biomarkers, and continued interdisciplinary collaborations integrating multi-omics, advanced imaging, and AI-driven analytics. Ultimately, decoding small EV-mediated communication networks could unlock new strategies to reprogram the TME, overcome therapeutic resistance, and improve patient outcomes in BC.

## Data Availability

No datasets were generated or analysed during the current study.

## Abbreviations

AI: Artificial intelligence
ASCs: Adipose-derived stem cells
BBB: Blood-brain barrier
BC: Breast cancer
CAF: Cancer-associated fibroblasts
Cav-1: Caveolin-1
CCT2: Chaperonin-containing TCP1 subunit 2
CDK: Cyclin-dependent kinase
CDK4/6i: CDK4/6 inhibitors
circRNAs: Circular RNAs
cirmiR-20a-5p: Circulating miR-20a-5p
c-Met: Mesenchymal-epithelial transition factor
CTLs: Cytotoxic T lymphocytes
DCs: Dendritic cells
DL: Deep learning
DNMT3A: DNA (cytosine-5)-methyltransferase 3 A
ECM: Extracellular matrix
EMT: Epithelial-to-mesenchymal transition
EphA2: EphrinA2
ER: Estrogen receptor
ESCRT: Endosomal Sorting Complex Required for Transport
EVs: Extracellular vesicles
ExoDS: Doxorubicin-encapsulated small EVs
FAK: Focal adhesion kinase
GMP: Good manufacturing practice
HER2: Human epidermal growth factor receptor 2
IBSP: Integrin-binding sialoprotein
ICAM1: Intercellular adhesion molecule 1
ILVs: Intraluminal vesicles
JAG1: Jagged-1
lncRNAs: long non-coding RNAs
MALAT1: LncRNA metastasis associated lung adenocarcinoma transcript 1
mDCs: Mature DCs
MDSCs: Myeloid-derived suppressor cells
Mef2c: Myocyte enhancer factor 2c
miRNAs: MicroRNAs
ML: Machine learning
mRNAs: Messenger RNAs
MVBs: Multivesicular bodies
NK: Natural killer
NKT: Natural killer T
NLP: Natural language processing
nSMase2: Neural sphingomyelinase 2
PBMCs: Peripheral blood mononuclear cells
PDT: Photodynamic therapy
PELI1: Pellino-1
PHD2: Prolyl hydroxylase domain 2
PR: Progesterone receptor
pre-miRNA: Precursor miRNA
pri-miRNA: Primary miRNA
RB1: Retinoblastoma 1
RISC: RNA-induced silencing complex
ROS: Reactive oxygen species
RPPH1: LncRNA Ribonuclease P RNA component H1
SLC9A1: Solute carrier family 9 member A1
SNHG1: Small nucleolar host gene 1
SOCS3: Suppressors of cytokine signaling 3
TAMs: Tumor-associated macrophages
TCR: T cell receptor
TDEs: Small EVs released by tumor cells
TGF-β: Transforming growth factor beta
Th: T helper cell
THBS1: Thrombospondin 1
TME: Tumor microenvironment
TNBC: Triple-negative breast cancer
Tregs: Regulatory T cells
